# Supplementary figures and images for: Expanded Genomic Sampling Refines Current Understanding of the Distribution and Evolution of Sulfur Metabolisms in the Desulfobulbales
Source: Front Microbiol. 2021 May 19;12:666052. doi: 10.3389/fmicb.2021.666052 (PMC8170396; doi:10.3389/fmicb.2021.666052)

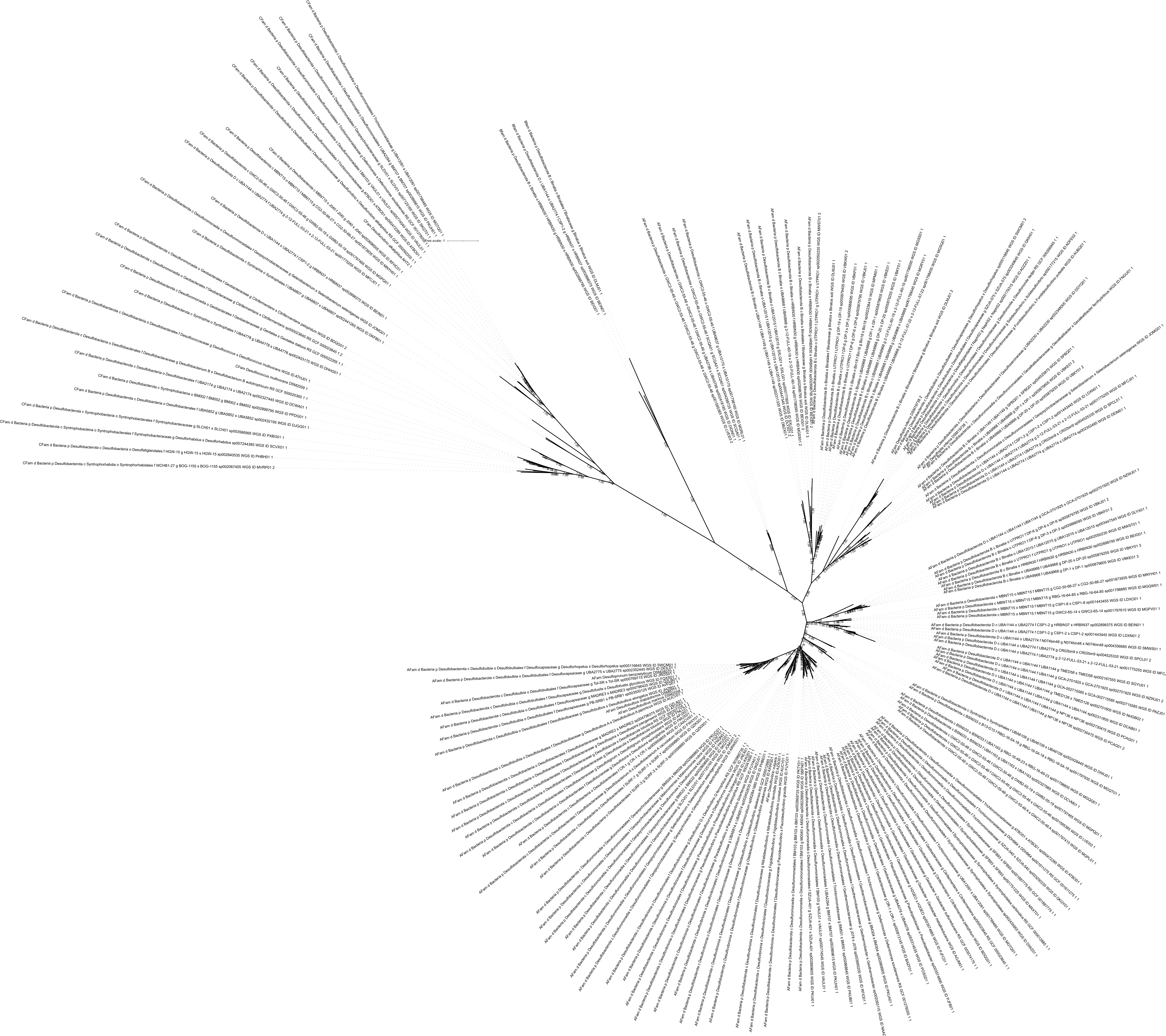

Supplement: Supplementary Figure 1 — Phylogeny of Heme-Copper Oxidoreductase (HCO) proteins from members of the Desulfobacterota. Leaves are labeled with the family of HCO ( A-, B-, or C-family) GTDB taxonomic assignments and WGS or Genbank IDs, nodes are labeled with TBE support value. [file Data_Sheet_1.PDF]

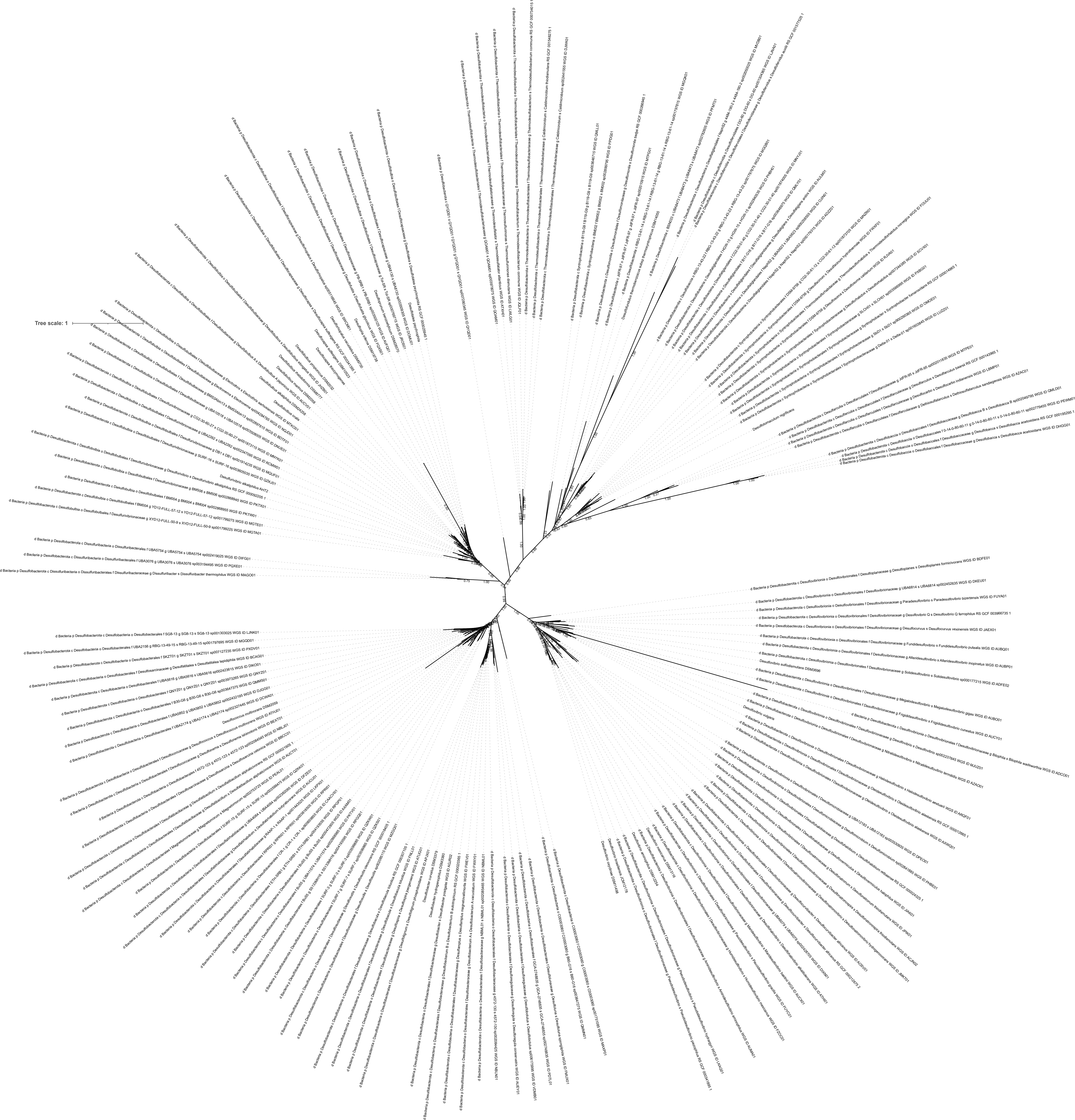

Supplement: Supplementary Figure 2 — Phylogeny of concatenated DsrA, DsrB, DsrC, AprA, and AprB proteins from members of the Desulfobacterota. Leaves are labeled with GTDB taxonomic assignments and WGS or Genbank IDs, nodes are labeled with TBE support value. [file Data_Sheet_2.PDF]

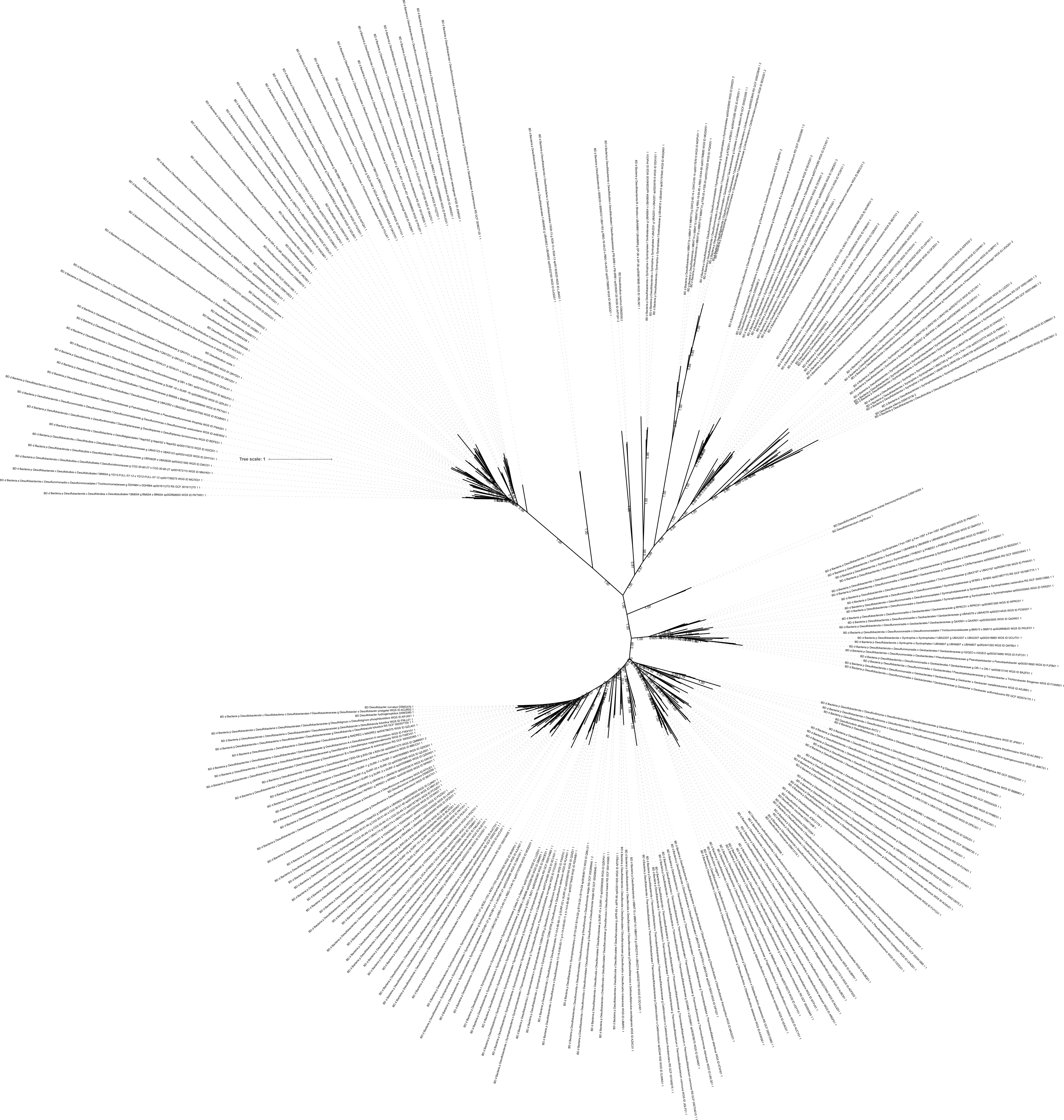

Supplement: Supplementary Figure 3 — Phylogeny of bd oxidase proteins from members of the Desulfobacterota. Leaves are labeled with GTDB taxonomic assignments and WGS or Genbank IDs, nodes are labeled with TBE support value. [file Data_Sheet_3.PDF]
