# Supplementary material for: Expanded Genomic Sampling Refines Current Understanding of the Distribution and Evolution of Sulfur Metabolisms in the Desulfobulbales
Source: Front Microbiol. 2021 May 19;12:666052. doi: 10.3389/fmicb.2021.666052 (PMC8170396; doi:10.3389/fmicb.2021.666052)
Supplement: Supplementary file 8 [file Data_Sheet_6.pdf]

Supplemental References

1. Böttcher, M. E., Thamdrup, B., Gehre, M., Theune, A. (2005) $^{34}S/^{32}S$ and $^{18}O/^{16}O$ Fractionation During Sulfur Disproportionation by Desulfobulbus propionicus *Geomicrobiology Journal* **Vol. 22(5), pp. 219-226** DOI: 10.1080/01490450590947751
2. Canfield, D. E., Thamdrup, B., Fleischer, S. (1998) Isotope fractionation and sulfur metabolism by pure and enrichment cultures of elemental sulfur disproportionating bacteria *Limnology and Oceanography* **Vol.43(2), pp. 253-264** DOI: 10.4319/lo.1998.43.2.0253
3. Cross, K. L., Chirania, P., Xiong, W., Beall, C. J., Elkins, J. G., Giannone, R. J., et al. (2018) Insights into the Evolution of Host Association through the Isolation and Characterization of a Novel Human Periodontal Pathobiont, *Desulfobulbus oralis*, *mBio* **Vol. 9(2)** DOI: 10.1128/mBio.02061-17
4. El Houari, A., Ranchou-Peyruse, M., Ranchou-Peyruse, A., Dakdaki, A., Guignard, M., Idouhammou, L., et al. (2017) *Desulfobulbus oligotrophicus* sp. nov., a sulfate-reducing and propionate-oxidizing bacterium isolated from a municipal anaerobic sewage sludge digester *International journal of systematic and evolutionary microbiology* **Vol. 67(2), pp. 275-281** DOI: 10.1099/ijsem.0.001615
5. Finster, K. (2008) Microbiological disproportionation of inorganic sulfur compounds *Journal of Sulfur Chemistry* **Vol. 29(3-4), pp. 281-292** DOI: 10.1080/17415990802105770
6. Finster, K., Liesack, W., Thamdrup, B. (1998) Elemental Sulfur and Thiosulfate Disproportionation by *Desulfocapsa sulfoexigens* sp. nov., a New Anaerobic Bacterium Isolated from Marine Surface Sediment *Applied and Environmental Microbiology* **Vol. 64(1), p. 119** DOI: 10.1128/AEM.64.1.119-125.1998
7. Fuseler, K., Cypionka, H. (1995) Elemental sulfur as an intermediate of sulfide oxidation with oxygen by Desulfobulbus propionicus *Archives of Microbiology* **164:104–109** DOI: 10.1007/BF02525315
8. Gittel, A., Seidel, M., Kuever, J., Galushko, A. S., Cypionka, H., Konneke1, M. (2010) *Desulfopila inferna* sp. nov., a sulfate-reducing bacterium isolated from the subsurface of a tidal sand-flat *International Journal of Systematic and Evolutionary Microbiology* **60, 1626–1630** DOI: 10.1099/ijs.0.015644-0
9. Isaksen, M. F., Teske, A. (1996) *Desulforhopalus vacuolatus* gen. nov., sp. nov., a new moderately psychrophilic sulfate-reducing bacterium with gas vacuoles isolated from a temperate estuary *Archives of Microbiology* **Vol. 166(3), pp. 160-168** DOI: 10.1007/s002030050371
10. Janssen, P. H., Schuhmann, A., Bak, F., Liesack, W. (1996) Disproportionation of inorganic sulfur compounds by the sulfate-reducing bacterium *Desulfocapsa thiozymogenes* gen. nov., sp. nov. *Archives of Microbiology* **Vol. 166(3), pp. 184-192** DOI: 10.1007/s002030050374
11. Junghare, M., Schink, B. (2015) *Desulfoprunum benzoelyticum* gen. nov., sp. nov., a Gram-stain-negative, benzoate-degrading, sulfate-reducing bacterium isolated from a wastewater treatment plant *International journal of systematic and evolutionary microbiology* **Vol. 65(Pt 1), pp. 77-84** DOI: 10.1099/ijs.0.066761-0
12. Knoblauch, C., Sahm, K., Jorgensen, B. (1999) Psychrophilic sulfate-reducing bacteria isolated from permanently cold Arctic marine sediments: description of *Desulfofrigus*

oceanense gen.nov., sp. nov., *Desulfofrigus fragile* sp. nov., *Desulfofaba gelida* gen. nov., sp. nov., *Desulfotalea psychrophila* gen. nov., sp. nov. and *Desulfotalea arctica* sp. nov *International Journal of Systematic Bacteriology* **Vol. 49(4), pp. 1631-1643** DOI: 10.1099/00207713-49-4-1631

13. Kramer, M., Cypionka, H. (1989) Sulfate formation via ATP sulfurylase in thiosulfate- and sulfite-disproportionating bacteria *Archives of Microbiology* **151:232–237** DOI: 10.1007/BF00413135

14. Kuever, J. (2014) The Family *Desulfobulbaceae*. In: Rosenberg, E., DeLong, E. F., Lory, S., Stackebrandt, E., Thompson, F., (eds) The Prokaryotes. Springer, Berlin, Heidelberg

15. Kuever, J., Rainey, F. A., Widdel, F. (2005) Genus I Desulfobulbus. In: Brenner DJ, Krieg NR, Staley JT, Garrity GM (eds) Bergey's manual of systematic bacteriology, vol 2, 2nd edn, (The Proteobacteria), part C (The Alpha-, Beta-, Delta-, and Epsilonproteobacteria) *Springer, New York*, **pp. 988–92**

16. Lie, T. J., Clawson, M. L., Godchaux, W., Leadbetter, E. R. (1999) Sulfidogenesis from 2-Aminoethanesulfonate (Taurine) Fermentation by a Morphologically Unusual Sulfate-Reducing Bacterium, *Desulforhopalus singaporensis* sp. nov. *Applied and Environmental Microbiology* **Vol. 65(8), p. 3328** DOI: 10.1128/AEM.65.8.3328-3334.1999

17. Lien, T., Madsen, M., Steen, I. H., Gjerdevik, K. (1998) *Desulfobulbus rhabdoformis* sp. nov., a sulfate reducer from a water–oil separation system *International Journal of Systematic Bacteriology* **48:469–474** DOI: 10.1099/00207713-48-2-469

18. Lovley, D. R., Phillips, E. J. P. (1994) Novel processes for anaerobic sulfate production from elemental sulfur by sulfate-reducing bacteria *Applied and Environmental Microbiology* **60:2394–2399**

19. Rosenberg, E. (Editor-in-Chief), DeLong, E. L., Lory, S., Stackebrandt, E., Thompson, F. (Eds.) (2014) The Prokaryotes – Deltaproteobacteria and Epsilonproteobacteria *Springer-Verlag Berlin Heidelberg* DOI: 10.1007/978-3-642-39044-9_267

20. Samain, E., Dubourguier, H. C., Albagnac, G. (1984) Isolation and characterisation of *Desulfobulbus elongatus* sp. nov. from a mesophilic industrial digester *Systematic and Applied Microbiology* **5:391–401** DOI: 10.1016/S0723-2020(84)80040-5

21. Sass, A., Rutter, H., Cypionka, H., Sass, H. (2002) *Desulfobulbus mediterraneus* sp. nov., a sulfate-reducing bacterium growing on mono- and disaccharides. *Archives of Microbiology* **177:468–474** DOI: 10.1007/s00203-002-0415-5

22. Sorokin, D. Y., Tourova, T. P., Panteleeva, A. N., Muyzer G. (2012) *Desulfonatronobacter acidivorans* gen. nov., sp. nov. and *Desulfobulbus alkaliphilus* sp. nov. haloalkaliphilic heterotrophic sulfate-reducing bacteria from soda lakes *International Journal of Systematic and Evolutionary Microbiology* **62:2107–2113** DOI: 10.1099/ijs.0.029777-0

23. Suzuki, D., Ueki, A., Amaishi, A., Ueki, K. (2007) *Desulfobulbus japonicus* sp. nov., a novel Gram-negative, propionate-oxidizing, sulfate-reducing bacterium isolated from an estuarine sediment in Japan *International Journal of Systematic and Evolutionary Microbiology* **57:849–855** DOI: 10.1099/ijs.0.64855-0

24. Suzuki, D., Ueki, A., Amaishi, A., Ueki, K. (2007) *Desulfopila aestuarii* gen. nov., sp. nov., a Gram-negative, rod-like,sulfate-reducing bacterium isolated from an estuarine sediment in

Japan *International Journal of Systematic and Evolutionary Microbiology* **Vol. 57(3), pp. 520-526** DOI: 10.1099/ijs.0.64600-0

25. Widdel, F. (1980) Anaerober Abbau von Fettsauren und Benzoesaure durch neu isolierte Arten sulfat-reduzierender Bakterien. Dissertation. Georg-August-Universitat zu Gottingen. Lindhorst/Schaumburg-Lippe, Gottingen, **pp. 7–149**

26. Widdel, F., Pfennig, N. (1982) Studies on dissimilatory sulfate-reducing bacteria that decompose fatty acids. II. Incomplete oxidation of propionate by *Desulfobulbus propionicus* gen. nov. sp. Nov. *Arch Microbiol* **131:360–365** DOI: 10.1007/BF00411187
