# Supplementary material for: Expanded Genomic Sampling Refines Current Understanding of the Distribution and Evolution of Sulfur Metabolisms in the Desulfobulbales
Source: Front Microbiol. 2021 May 19;12:666052. doi: 10.3389/fmicb.2021.666052 (PMC8170396; doi:10.3389/fmicb.2021.666052)
Supplement: Supplementary Figure 4 — Phylogeny of DsrA proteins from members of the Desulfobacterota. Leaves are labeled with GTDB taxonomic assignments and WGS or Genbank IDs, nodes are labeled with TBE support value. [file Data_Sheet_4.PDF]

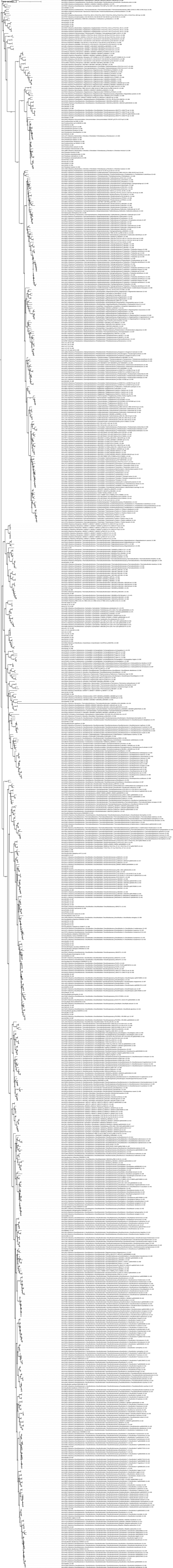
Tree scale: 1
dsrA DDYS d Bacteria p Deferribacterota c Deferribacteres o UBA228 f UBA228 g UBA228 s UBA228 sp1 1/1-390
dsrA MDSU d Bacteria p Campylobacterota c Desulfurellia o Desulfurellales f Desulfurellaceae g Desulfurella s Desulfurella amilsii 1/1-398
dsrA DKBB d Bacteria p Acidobacteriota c UBA6911 o UBA6911 f UBA6911 g UBA6911 s 1/1-379
dsrA PMAY d Bacteria p Desulfobacterota c Syntrophia o Syntrophales f Fen-1087 g Fen-1087 s Fen-1087 sp003161855 1/1-379
dsrA OKRB01 1/1-376
dsrA NCBR d Bacteria p Acidobacteriota c Thermoanaerobaculia o Thermoanaerobaculales f Thermoanaerobaculaceae g RBG-13-68-16 s RBG-13-68-16 sp1 1/1-381
dsrA MEMX d Bacteria p Myxococcota c Myxococcia o Myxococcales f Anaeromyxobacteraceae g Anaeromyxobacter s 1/1-386
dsrA J133 1/1-388
dsrA MGST d Bacteria p Myxococcota c XYA12-FULL-58-9 o XYA12-FULL-58-9 f XYA12-FULL-58-9 g XYA12-FULL-58-9 s XYA12-FULL-58-9 sp1 1/1-390
dsrA PFKP d Bacteria p Armatimonadota c CG2-30-59-28 o CG2-30-59-28 f CG2-30-66-41 g CG2-30-66-41 s 1/1-384
dsrA PGXB d Bacteria p Zixibacteria c MSB-5A5 o Zixibacteria-1 f Zixibacteria-1 g Zixibacteria-1 s 1/1-386
dsrA MGNC01 1/1-386
dsrA RCNO01 1/1-384
dsrA RCNP01 1/1-391
dsrA RCNQ01 1/2-387
dsrA MHAY d Bacteria p Bacteroidota c Kapabacteria o Kapabacteriales f XYC2-FULL-35-21 g XYC2-FULL-35-21 s 1/2-387
dsrA MGZV d Bacteria p Bacteroidota c Kapabacteria o Kapabacteriales f XYC2-FULL-35-21 g XYC2-FULL-35-21 s 1/2-387
dsrA MHAN d Bacteria p Bacteroidota c Kapabacteria o Kapabacteriales f XYC2-FULL-35-21 g XYC2-FULL-35-21 s 1/2-387
dsrA MHYH d Bacteria p Planctomycetota c Planctomycetes o Pirellulales f Thermoguttaceae g RBG-13-63-9 s 1/1-382
dsrA MFNB d Bacteria p SAR324 c SAR324 o XYD2-FULL-50-16 f XYD2-FULL-50-16 g XYC1-FULL-56-13 s 1/1-390
dsrA MFNE d Bacteria p SAR324 c SAR324 o XYD2-FULL-50-16 f XYD2-FULL-50-16 g XYD2-FULL-50-16 s 1/1-402
dsrA MNYV d Bacteria p CG2-30-53-67 c CG2-30-53-67 o CG2-30-53-67 f CG2-30-53-67 g CG2-30-53-67 s 3/1-397
dsrA PFFA d Bacteria p CG2-30-53-67 c CG2-30-53-67 o CG2-30-53-67 f CG2-30-53-67 g CG2-30-53-67 s 3/1-397
dsrA PFUI d Bacteria p UBA9089 c CG2-30-40-21 o CG2-30-40-21 f CG2-30-40-21 g CG2-30-40-21 s 1/1-380
dsrA PCSH d Bacteria p UBA9089 c CG2-30-40-21 o CG2-30-40-21 f CG2-30-40-21 g CG2-30-40-21 s 1/1-380
dsrA MELI d Bacteria p Actinobacteriota c UBA9087 o UBA10029 f UBA10029 g UBA10029 s 1/1-382
dsrA PFKS d Bacteria p Actinobacteriota c UBA9087 o UBA10029 f UBA10029 g CG2-30-50-142 s 1/1-378
dsrA PFFU d Bacteria p Actinobacteriota c UBA9087 o UBA10029 f UBA10029 g CG2-30-50-142 s 1/1-378
dsrA MHDL01 d Bacteria p Nitrospinota c UBA9942 o UBA9942 f UBA9942 g PLOWO2-12-39-15 s 1/1-384
dsrA MHDO01 d Bacteria p Nitrospinota c UBA9942 o UBA9942 f UBA9942 g UBA9942 s 1/1-384
dsrA MHDO d Bacteria p Nitrospinota c UBA9942 o UBA9942 f UBA9942 g UBA9942 s 1/1-384
dsrA MHEL01 d Bacteria p Nitrospirota c Nitrospiria o 9FT-COMBO-42-15 f 9FT-COMBO-42-15 g 9FT-COMBO-42-15 s 1/1-387
dsrA MNYV d Bacteria p CG2-30-53-67 c CG2-30-53-67 o CG2-30-53-67 f CG2-30-53-67 g CG2-30-53-67 s 1/1-388
dsrA PFFA d Bacteria p CG2-30-53-67 c CG2-30-53-67 o CG2-30-53-67 f CG2-30-53-67 g CG2-30-53-67 s 1/1-388
dsrA DLRR01 d Bacteria p Nitrospinota c UBA7883 o UBA7883 f UBA7883 g UBA7883 s 1/1-391
dsrA DLRR d Bacteria p Nitrospinota c UBA7883 o UBA7883 f UBA7883 g UBA7883 s 1/1-391
dsrA MGZB d Bacteria p Proteobacteria c Gammaproteobacteria o Chromatiales f Sedimenticolaceae g YD12-FULL-61-37 s 2/1-384
dsrA MGYZ d Bacteria p Proteobacteria c Gammaproteobacteria o Chromatiales f Sedimenticolaceae g YD12-FULL-61-37 s 2/1-384
dsrA PEAJ d Bacteria p Proteobacteria c Magnetococcia o Magnetococcales f DC0425bin3 g HA3dbin3 s 1/1-385
dsrA PDZU d Bacteria p Proteobacteria c Magnetococcia o Magnetococcales f WMHbin3 g WMHbinv6 s 1/1-384
dsrA PEAG d Bacteria p Proteobacteria c Magnetococcia o Magnetococcales f WMHbin3 g HCHbin5 s 3/1-384
dsrA PDZX d Bacteria p Proteobacteria c Magnetococcia o Magnetococcales f WMHbin3 g WMHbin3 s 1/1-408
dsrA PEAM d Bacteria p Proteobacteria c Magnetococcia o Magnetococcales f UBA8363 g GCA-2753565 s 2/1-386
dsrA PDZY d Bacteria p Proteobacteria c Magnetococcia o Magnetococcales f UBA8363 g UBA8363 s 1/1-384
dsrA PEAN d Bacteria p Proteobacteria c Magnetococcia o Magnetococcales f UBA8363 g UBA8363 s 1/1-384
dsrA PEAK d Bacteria p Proteobacteria c Magnetococcia o Magnetococcales f UBA8363 g UBA8363 s UBA8363 sp2 1/1-384
dsrA PEAI d Bacteria p Proteobacteria c Magnetococcia o Magnetococcales f UBA8363 g UBA8363 s UBA8363 sp2 1/1-384
dsrA MHEK01 d Bacteria p Nitrospirota c RBG-16-64-22 o RBG-16-64-22 f RBG-16-64-22 g RBG-16-64-22 s 1/1-400
dsrA MHEK d Bacteria p Nitrospirota c RBG-16-64-22 o RBG-16-64-22 f RBG-16-64-22 g RBG-16-64-22 s 1/1-400
dsrA DKAT d Bacteria p Spirochaetota c UBA6919 o UBA6919 f UBA6919 g UBA6919 s 1/1-386
dsrA PEAG d Bacteria p Proteobacteria c Magnetococcia o Magnetococcales f WMHbin3 g HCHbin5 s 1/1-392
dsrA LVJN d Bacteria p Proteobacteria c Magnetococcia o Magnetococcales f Magnetococcaceae g Magnetofaba s Magnetofaba australis 1/1-403
dsrA PEAM d Bacteria p Proteobacteria c Magnetococcia o Magnetococcales f UBA8363 g GCA-2753565 s 1/1-388
dsrA DCWP d Bacteria p SAR324 c SAR324 o SAR324 f NAC60-12 g UBA1014 s UBA1014 sp1 1/1-384
dsrA NYYL d Bacteria p Proteobacteria c Alphaproteobacteria o Rhodospirillales f 2-02-FULL-58-16 g GCA-2686765 s 1/1-384
dsrA DCWS d Bacteria p Desulfobacterota c Desulfobacteria o Desulfobacterales f UBA2156 g UBA2156 s 1/1-386
dsrA DCWS d Bacteria p Desulfobacterota c Desulfobacteria o Desulfobacterales f UBA2156 g UBA2156 s UBA2156 sp002328165 1/1-386
dsrA SWCM01 1/1-384
dsrA SWCN01 1/1-384
dsrA DNSP01 1/1-386
dsrA MAXK d Bacteria p Desulfobacterota c Desulfobulbia o Desulfobulbales f Desulfocapsaceae g UBA2775 s UBA2775 sp1 1/1-384
dsrA MAXQ d Bacteria p Desulfobacterota c Desulfobulbia o Desulfobulbales f Desulfocapsaceae g UBA2775 s UBA2775 sp1 1/1-384
dsrA MAXH d Bacteria p Desulfobacterota c Desulfobulbia o Desulfobulbales f Desulfocapsaceae g UBA2775 s UBA2775 sp001750925 1/1-384
