# Supplementary material for: Expanded Genomic Sampling Refines Current Understanding of the Distribution and Evolution of Sulfur Metabolisms in the Desulfobulbales
Source: Front Microbiol. 2021 May 19;12:666052. doi: 10.3389/fmicb.2021.666052 (PMC8170396; doi:10.3389/fmicb.2021.666052)
Supplement: Supplementary Figure 5 — Phylogeny of AprA proteins from members of the Desulfobacterota. Leaves are labeled with GTDB taxonomic assignments and WGS or Genbank IDs, nodes are labeled with TBE support value. [file Data_Sheet_5.PDF]

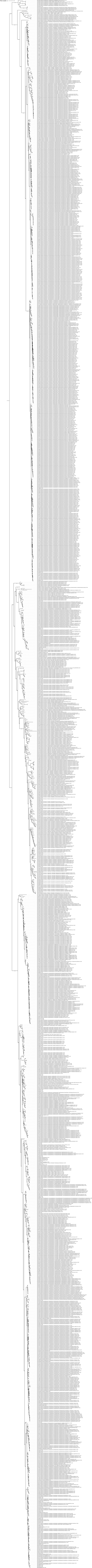
Tree scale: 1
