# Supplementary material for: Expanded Genomic Sampling Refines Current Understanding of the Distribution and Evolution of Sulfur Metabolisms in the Desulfobulbales
Source: Front Microbiol. 2021 May 19;12:666052. doi: 10.3389/fmicb.2021.666052 (PMC8170396; doi:10.3389/fmicb.2021.666052)
Supplement: Supplementary Table 1 — Summary of capacity to perform microbial sulfate reduction (type of electron acceptor used indicated) and/or microbial sulfur disproportionation (type of disproportionation performed indicated) as described in peer-reviewed reports. NR, not reported; NA, not applicable; (1) Only “personal communication” found then, by default, indicated as Not Reported; (2) Reported in the reference given but no supporting study found. [file Table_1.docx]

|  |  | **Ref.** | NA | NA | NA | NA | NA | [9] | NA | NA | [21] | [1, 7, 13, 18, 26] | [6] | [2, 10] | [5] | NA | NA | [12, 19] | NA |
| --- | --- | --- | --- | --- | --- | --- | --- | --- | --- | --- | --- | --- | --- | --- | --- | --- | --- | --- | --- |
|  |  | **S_2_O_3_^2-^ disp.** | NR | NR | NR | NR | NR | N | NR | NR | N | Y | Y | Y | N | NR | NR | N | NR |
|  |  | **Ref.** | NA | NA | NA | NA | NA | NA | NA | NA | [21] | [4] | [6] | [2, 10] | [5] | NA | NA | NA | NA |
|  |  | **SO_3_^2-^ disp.** | NR | NR | NR | NR | NR | NR | NR | NR | N | N | Y | Y | N | NR | NR | NR | NR |
|  |  | **Ref.** | NA | NA | NA | NA | NA | [9] | [10] | NA | NA | [1, 10] | [6] | [2, 10] | [5] | NA | [19] | [12, 19] | NA |
|  | **MSD** | **S^0^ disp.** | NR | NR | NR (1) | NR | NR | N | N | NR | NR | Y | Y | Y | Y | NR | Y (2) | N | NR |
|  |  | **Ref.** | [8] | [14, 15, 23] | [22] | [11] | [17] | [9] | [20] | [ 23] | [21] | [22, 25, 26] | [6] | [11, 19] | [11] | [11, 19, 24] | [11, 16] | [19] | [3] |
| **Metabolism** | **SRM** |  | SO_4_^2-^ and SO_3_^2-^ reduction, no S_2_O_3_^2-^ reduction | SO_4_^2-^, SO_3_^2-^, S_2_O_3_^2-^ reduction | SO_4_^2-^ and SO_3_^2-^ reduction, no S_2_O_3_^2-^ reduction | SO_4_^2-^ and SO_3_^2-^ reduction, no S_2_O_3_^2-^ reduction | SO_4_^2-^, SO_3_^2-^, S_2_O_3_^2-^ reduction | SO_4_^2-^, SO_3_^2-^, S_2_O_3_^2-^ reduction | SO_4_^2-^, SO_3_^2-^, S_2_O_3_^2-^ reduction | SO_4_^2-^ and S_2_O_3_^2-^ reduction, no SO_3_^2-^ reduction | SO_4_^2-^, SO_3_^2-^, S_2_O_3_^2-^ reduction | SO_4_^2-^, SO_3_^2-^,  S_2_O_3_^2-^ reduction | N | SO_4_^2-^, SO_3_^2-^, S_2_O_3_^2-^ reduction | SO_4_^2-^, SO_3_^2-^, S_2_O_3_^2-^ reduction | SO_4_^2-^, SO_3_^2-^, S_2_O_3_^2-^ reduction | SO_4_^2-^, SO_3_^2-^, S_2_O_3_^2-^ reduction | SO_4_^2-^, SO_3_^2-^, S_2_O_3_^2-^ reduction | SO_4_^2-^ and S_2_O_3_^2-^ reduction, no SO_3_^2-^ reduction |
| **Species** |  |  | *Desulfopila inferna* | *Desulfobulbus marinus* | *Desulfobulbus alkaliphilus* | *Desulfoprunum benzoelyticum* | *Desulfobulbus rhabdoformis* | *Desulforhopalus vacuolatus* | *Desulfobulbus elongatus* | *Desulfobulbus japonicus* | *Desulfobulbus mediterraneus* | *Desulfobulbus propionicus* | *Desulfocapsa sulfexigens* | *Desulfocapsa thiozymogenes* | *Desulfofustis glycolicus* | *Desulfopila aestuarii* | *Desulforhopalus singaporensis* | *Desulfotalea psychriphila* | *Desulfobulbus oralis/1-2189* |
